# Na/Cu‐Doping Engineering of Carbon Nitride for High‐Performance Visible‐Light Degradation of Iopamidol

**DOI:** 10.1002/smsc.70341

**Published:** 2026-07-08

**Authors:** Samuel Ashu Abey, Carl Fernandes, Emma Emanuelsson, Nuno M. Reis, Antonio J. Expósito

**Affiliations:** ^1^ Department of Chemical Engineering University of Bath Claverton Down UK; ^2^ Centre for Integrated Materials, Processes & Structures (IMPS) University of Bath Claverton Down UK; ^3^ Centre for Regenerative Design & Engineering for a Net Positive World (RENEW) University of Bath Claverton Down UK; ^4^ Centre for Bioengineering and Biomedical Technologies (CBio) University of Bath Claverton Down UK

**Keywords:** catalyst doping, graphitic carbon nitride, Iopamidol, pollutant degradation, visible‐light catalysis

## Abstract

Visible‐light‐driven photocatalytic generation of hydroxyl radicals provides a sustainable pathway for the photodegradation of emerging contaminants. Particularly, carbon nitride (g‐C_3_N_4_) is a promising photocatalyst for the removal of emerging contaminants; however, it suffers from a limited surface area and a bandgap that restricts its visible‐light photocatalytic efficiency. To overcome these limitations, this study presents a novel copper–sodium codoped graphitic carbon nitride (NaCu‐g‐C_3_N_4_) photocatalyst with enhanced visible‐light photocatalytic activity tested for the degradation of Iopamidol, a persistent and toxic pollutant resistant to conventional wastewater treatment plants. The codoping of Na and Cu was strategically employed to reduce the bandgap energy, enhance visible‐light absorption, and increase the surface area, thereby improving the photocatalytic activity of carbon nitride. When combined with the oxidizing agent H_2_O_2_, the system accelerated the formation of oxidizing radicals. The synergy of NaCu‐g‐C_3_N_4_ and H_2_O_2_ yielded 82.3% degradation of Iopamidol within 120 min under visible light at pH 3, marking the highest and first successful reported visible‐light‐driven degradation of Iopamidol. These findings highlight the potential of NaCu‐g‐C_3_N_4_ as a cost‐effective semiconductor photocatalyst for the efficient and sustainable removal of persistent pollutants.

## Graphical abstract

Schematic representation of doped carbon nitride photocatalysts and their benefits in promoting light absorption, charge separation, and reactive species generation for efficient Iopamidol degradation.

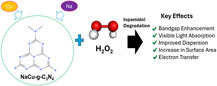

## 1 Introduction

Pharmaceutical waste, including persistent and potentially toxic iodinated X‐ray contrast agents used in medical imaging of blood vessels, is increasingly contaminating water bodies and posing significant risks to both human health and the environment [1]. It is therefore necessary to develop environmentally friendly and effective water purification methods for the oxidation of micropollutants. In particular, Iopamidol, an iodinated contrast agent used for computerized tomography and other radiological tests, has been found in wastewater treatment plant effluents and surface waters [2]. Iopamidol's presence in source waters may lead to the formation of highly toxic iodinated disinfection by‐products [3]. In this context, visible‐light‐operating semiconductor photocatalysts have garnered interest for use in water treatment processes [4, 5]. Previous studies have explored the degradation of Iopamidol using advanced oxidation processes, including UV/H_2_O_2_, UV/persulfate, and UV/chlorine systems [6]. Other studies have reported on the use of photocatalysis, including UV/sulfite, UV/TiO_2_, and UV/NH_2_Cl, to remove Iopamidol in wastewater [7, 8]. All previous research on the removal of Iopamidol has focused on UV‐based methods; however, more sustainable approaches are desirable, particularly those utilizing visible‐light degradation.

Previous studies have highlighted photocatalysts that use visible light to purify water sustainably and efficiently [9, 11]. Recently, graphitic carbon nitride (g‐C_3_N_4_), a metal‐free polymer n‐type semiconductor, has sparked interest in the photocatalytic degradation of micropollutants using visible light [12]. Despite its chemical stability and nontoxicity, the material still faces limitations, including low specific surface area, an inadequate bandgap, and limited visible‐light activity [13, 14]. The photocatalytic performance of g‐C_3_N_4_ can be enhanced by increasing surface area, providing more active sites for photocatalytic reactions, and bandgap alterations [15]. Several works have attempted to overcome g‐C_3_N_4_ limitations in terms of surface area and visible‐light absorption through techniques such as exfoliation into nanosheets, thermal etching, and bandgap reduction via doping metals and nonmetals [16, 17]. Although metals, such as Na, Pt, Au, and K, have been used as dopants to act as electron traps, reduce electron–hole recombination, and modify bandgaps to enhance photocatalytic performance, conventional doping strategies still exhibit limitations in charge carrier transfer, surface area, and bandgap modulation [18, 25].

To address these shortcomings and opportunities, this study explores the codoping of g‐C_3_N_4_ with Na and Cu for visible‐light photodegradation of Iopamidol, leveraging the complementary roles of these ions in enhancing charge separation and overall photocatalytic activity. Na can introduce shallow donor states, improving charge mobility, while Cu, with its variable oxidation states, facilitates efficient electron transfer and extends visible‐light absorption [26, 28]. This synergistic effect makes Na and Cu suitable candidates for codoping in g‐C_3_N_4_, ultimately optimizing its photocatalytic performance. The photocatalytic activity was further enhanced through the addition of H_2_O_2_, known to enhance the generation of radicals, thereby further improving photocatalytic degradation efficiency [29, 30]. H_2_O_2_ acts as an electron acceptor, facilitating the formation of hydroxyl radicals, which are highly reactive species in pollutant degradation [31]. Given these advantages, combining codoping strategies with H_2_O_2_‐assisted radical generation presents a promising approach for enhancing photocatalytic performance.

This study proposes a strategy of Na and Cu codoping in graphitic carbon nitride, along with H_2_O_2_‐assisted radical generation, to synergistically enhance dispersion, electron transfer, bandgap modulation, light absorption, and reactive species formation for improved and sustainable photocatalytic water treatment. To the best of our knowledge, this is the first study demonstrating the effective photocatalytic performance of this codoped catalyst, applied to achieve the first documented visible‐light‐driven degradation of Iopamidol, enabled by the effect of metal codoping.

## 2 Materials and Methods

### 2.1 Reagents

Nitric acid (HNO_3_) was purchased from Fisher Scientific (Loughborough, UK), copper sulfate (CuSO_4_) and hydrogen peroxide (H_2_O_2_) from Sigma‐Aldrich (Gillingham, UK). Sodium chloride (NaCl), dicyandiamide (C_2_H_4_N_4_), Iopamidol (C_17_H_22_I_3_N_3_O_8_), and hydrochloric acid (HCl) were sourced from Thermo Fisher Scientific (Loughborough, UK), and sodium hydroxide (NaOH) from Alfa Aesar (Heysham, UK). All chemicals were used as supplied without any further purification.

### 2.2 Synthesis of g‐C_3_N_4_ and NaCu‐g‐C_3_N_4_ Photocatalysts

**FIGURE 1:**
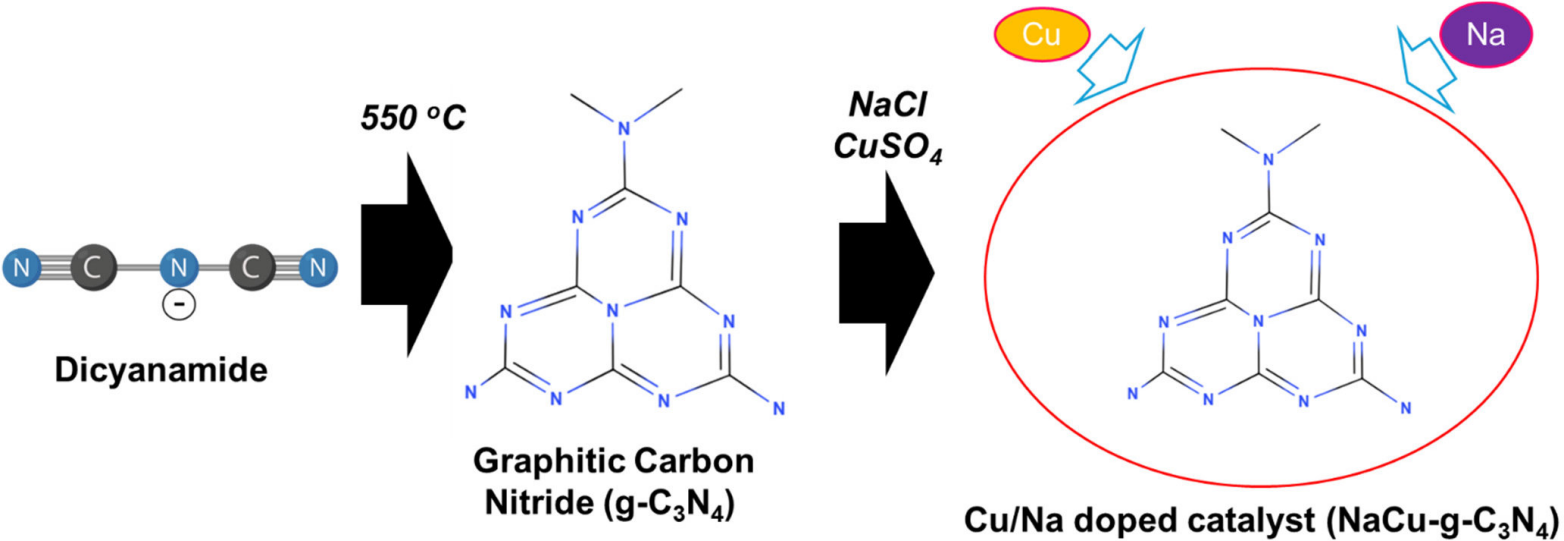
Schematic of synthesis process of g‐C_3_N_4_ and NaCu‐g‐C_3_N_4_. g‐C_3_N_4_ was synthesized from Dicyanamide at 550°C. NaCu‐g‐C_3_N_4_ was synthesized by wet impregnation using NaCl and CuSO_4_.

Graphitic carbon nitride (g‐C_3_N_4_) and NaCu‐g‐C_3_N_4_ were prepared using a methodology briefly shown in Figure 1. g‐C_3_N_4_ was synthesized by thermal degradation of dicyandiamide in a semiclosed crucible placed in a muffle furnace with an air environment. The equipment was programmed to heat up at a rate of 2°C min^−1^ to 450°C, which was maintained for 2 h. The temperature was then increased to 550°C at 2°C min^−1^ and kept for a further 4 h. After cooling, the material was washed with ultrapure water, filtered to eliminate any soluble by‐products, and dried overnight at 100°C. The thermal posttreatment of g‐C_3_N_4_ was carried out at 500°C for 3 h, and the resultant g‐C_3_N_4_ was codoped with NaCl and CuSO_4_ precursor salts by adapting the synthesis methods previously reported by Nisha et al. and Zhang et al. [28, 32]. Briefly, this started by dissolving 0.06 g of CuSO_4_ in 3 mL of water under stirring at 900 rpm and 20°C. A 1.0‐mL aliquot of this solution was then added to 1 g of carbon nitride in a crucible, which was sealed and heated at 80°C for 24 h. The resulting powder was then calcined in a muffle furnace at 550°C for 3 h, washed sequentially with HNO_3_ and deionized water under vacuum filtration, and dried at 40°C for 12 h before being ground into a fine powder. Separately, a 0.1 mol L^−1^ NaCl solution (0.29 g NaCl in 50 mL water) was prepared and slowly mixed with a solution of 10 g dicyandiamide in 75 mL water, stirred at 70°C for 1 h. The mixture was dried at 80°C for 24 h, ground, calcined at 550°C for 2 h, washed with boiling deionized water, and dried at 40°C for 12 h. For codoped synthesis, a 1.0 g sodium‐doped carbon nitride replaced carbon nitride in the copper doping procedure.

### 2.3 Molecular and Surface Characterization of the g‐C_3_N_4_ and NaCu‐g‐C_3_N_4_ Photocatalysts

X‐ray photoelectron spectroscopy (XPS) was performed using an ESCALAB250 spectrometer (Thermo Fisher Scientific, Waltham, MA, USA) to determine the elemental composition, chemical states, and electronic structure of the surface. Optical properties, including bandgap energy, were evaluated using ultraviolet–visible diffuse reflectance spectroscopy (UV–Vis DRS) on a Shimadzu UV‐3600 spectrophotometer, which facilitated the analysis of light absorption characteristics across the UV–Vis spectrum. The bandgap of a photocatalyst was analyzed via a Tauc plot. First, the photocatalyst's diffuse reflectance data were collected and converted into the absorption coefficient (*α*) using the Kubelka–Munk function. The photon energy (*hν*) was calculated from the wavelength of the incident light. For a Tauc plot, (*αhν*)^*n*^ was plotted against *hν*, where *n* = 2.0 for direct bandgap or *n* = 0.5 for indirect bandgap materials. The linear region of the plot was extrapolated to intersect the *x*‐axis, yielding the bandgap energy (*E*_g_). Scanning electron microscopy (SEM) images were captured using a JSM 7900F microscope (JEOL Ltd) after sputter coating the samples with gold. Elemental distribution and surface composition were further analyzed with energy‐dispersive X‐ray spectroscopy (EDX), employing two detectors (Ultim Max 170 mm^2^ and Ultim Extreme 100 mm^2^) at low accelerating voltages for high spatial resolution.

The Brunauer–Emmett–Teller (BET) method was utilized to evaluate the specific surface area of the samples. After degassing the materials at 150°C for 4 h under vacuum to remove adsorbed impurities, measurements were conducted at 77 K using a 3Flex instrument (Micromeritics Instruments, USA). Nitrogen adsorption–desorption isotherms were analyzed in the relative pressure range of *P*/*P_0_* = 0.1–0.4 to calculate surface area and pore characteristics. X‐ray diffraction (XRD) analysis was carried out with a diffractometer equipped with Mythen detectors, using Cu‐K*α*_1_ radiation (*λ* = 1.5406 Å) to identify crystalline phases and assess structural integrity. Patterns were recorded in continuous mode with a scanning duration of 2000 s at an operating voltage and current of 40 kV and 40 mA, respectively. Fourier‐transform infrared (FTIR) spectroscopy was employed to identify functional groups, molecular structure, and chemical bonds within the materials.

### 2.4 Degradation of Iopamidol Using Visible Light

Photodegradation experiments of Iopamidol were carried out under visible‐light irradiation (450‐W light‐emitting diode (LED) Lamp) by suspending up to 20 mg of NaCu‐g‐C_3_N_4_ in a 100 mL solution containing 5 mg L^−1^ Iopamidol dissolved in deionized water. While Iopamidol concentrations in natural environments are typically low, we employed higher concentrations in our experiments to establish a method for assessing the catalyst's performance and to demonstrate its capability to remove this pollutant under visible‐light irradiation. The suspension was stirred in the dark for 30 min to achieve adsorption–desorption equilibrium. Subsequently, 5 mM H_2_O_2_ was added simultaneously with the activation of the LED light source, and 3‐mL aliquots of the suspension were withdrawn at regular time intervals. The photocatalyst was separated from the suspension by passing the aliquots through a 0.45‐µm membrane filter to ensure accurate measurement of the remaining Iopamidol concentration in the solution.

Iopamidol concentration in the supernatant was determined using HPLC with a reversed‐phase C18 column (4.6 × 250 mm, 5 µm) and a mobile phase of methanol and 0.1% acetic acid water in a 10:90 (V/V) ratio [7]. The injection volume was 25 μL. The chromatographic column was set at 35°C, and the UV detector's wavelength was 242 nm. The influence of solution pH on photodegradation was tested in the pH range from 3.0 to 12.0, with the starting pH adjusted by 0.05 M HCl and 0.05 M NaOH solutions. The impact of catalyst dosage was investigated using varying quantities of NaCu‐g‐C_3_N_4_ (0–20 mg).

## 3 Results and Discussion

### 3.1 Synthesis and Characterization of g‐C_3_N_4_ and NaCu‐g‐C_3_N_4_ Photocatalysts

**FIGURE 2:**
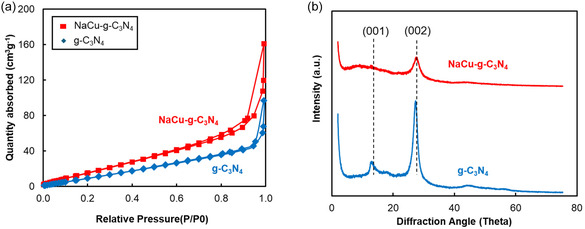
BET Surface area analysis of the g‐C_3_N_4_ and NaCu‐g‐C_3_N_4_. (a) Isotherm of N_2_ adsorption of g‐C_3_N_4_ and NaCu‐g‐C_3_N_4_; (b) XRD spectrum of g‐C_3_N_4_ and NaCu‐g‐C_3_N_4_.

We have carried out extensive molecular and surface characterization that showed doping of g‐C_3_N_4_ with Na and Cu yielded significant changes in the lattice of the photocatalyst particles, with benefits in terms of enhancement of surface area and improvement of photocatalytic activity. Both NaCu‐g‐C_3_N_4_ and g‐C_3_N_4_ materials synthesized demonstrated mesoporous characteristics and type IV adsorption isotherms (Figure 2a) [33]. Mesoporous structures provide an optimal balance between high surface area and good mass transport properties, enabling efficient diffusion of reactant molecules into the catalyst's pores [34]. NaCu‐g‐C_3_N_4_ showed a specific surface area about 38% larger compared with the undoped g‐C_3_N_4_ material, being 85.9 and 65.8 m^2^ g^−1^, respectively. The increase in surface area is presumably linked to the co‐doping, which introduces distortions in the crystal lattice of the photocatalyst, effectively increasing the specific surface area [35]. Furthermore, codoping is known to modify the stacking arrangement of layers in materials, resulting in thinner layers or partial exfoliation, exposing more surface area [36]. The enhanced surface area of NaCu‐g‐C_3_N_4_ allows for greater generation and diffusion of reactive species like hydroxyl radicals, crucial for breaking down organic pollutants [37]. Also, higher surface areas facilitate better dispersion of the NaCu‐g‐C_3_N_4_ catalyst in the reaction medium, ensuring uniform interaction with light and pollutants [38].

XRD revealed the diffraction peaks for g‐C_3_N_4_ and NaCu‐g‐C_3_N_4_ as shown in Figure 2b. The XRD spectra for g‐C_3_N_4_ exhibited two prominent peaks at 13° and 27°, corresponding to the (001) and (002) planes of g‐C_3_N_4_, respectively [39]. These planes are due to the repeating units of the tri‐s‐triazine ring of the g‐C_3_N_4_ [40]. Compared with bulk g‐C_3_N_4_, the disappearance of the (001) peak in NaCu‐g‐C_3_N_4_ suggests that Na/Cu codoping distorted the crystallinity of g‐C_3_N_4_, with Na atoms occupying nitrogen‐rich cavities and donating electrons to the surrounding C–N network, thereby inducing structural distortion and lattice relaxation [41, 42]. Furthermore, Cu incorporation introduces additional defects and further disrupts the C–N framework [43]**.** The broadening of the (002) reflection peak in NaCu‐g‐C_3_N_4_ indicates strain‐induced lattice distortion, confirming that Na/Cu codoping weakens the interlayer stacking of the tri‐s‐triazine units of g‐C_3_N_4_ [44, 45].

**FIGURE 3:**
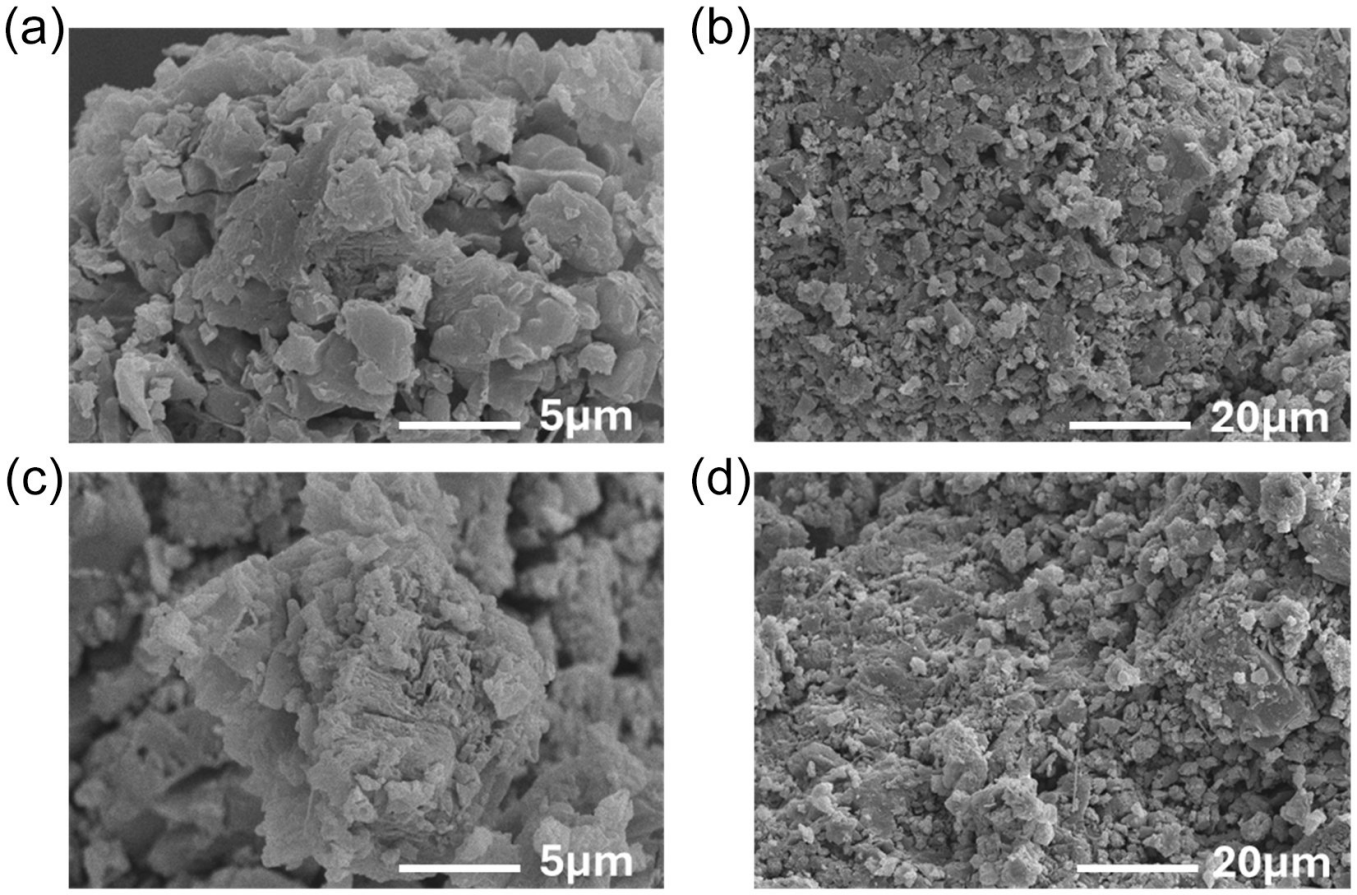
SEM images of (a,b) g‐C_3_N_4_ and (c,d) NaCu‐g‐C_3_N_4_ at different magnifications.

**FIGURE 4:**
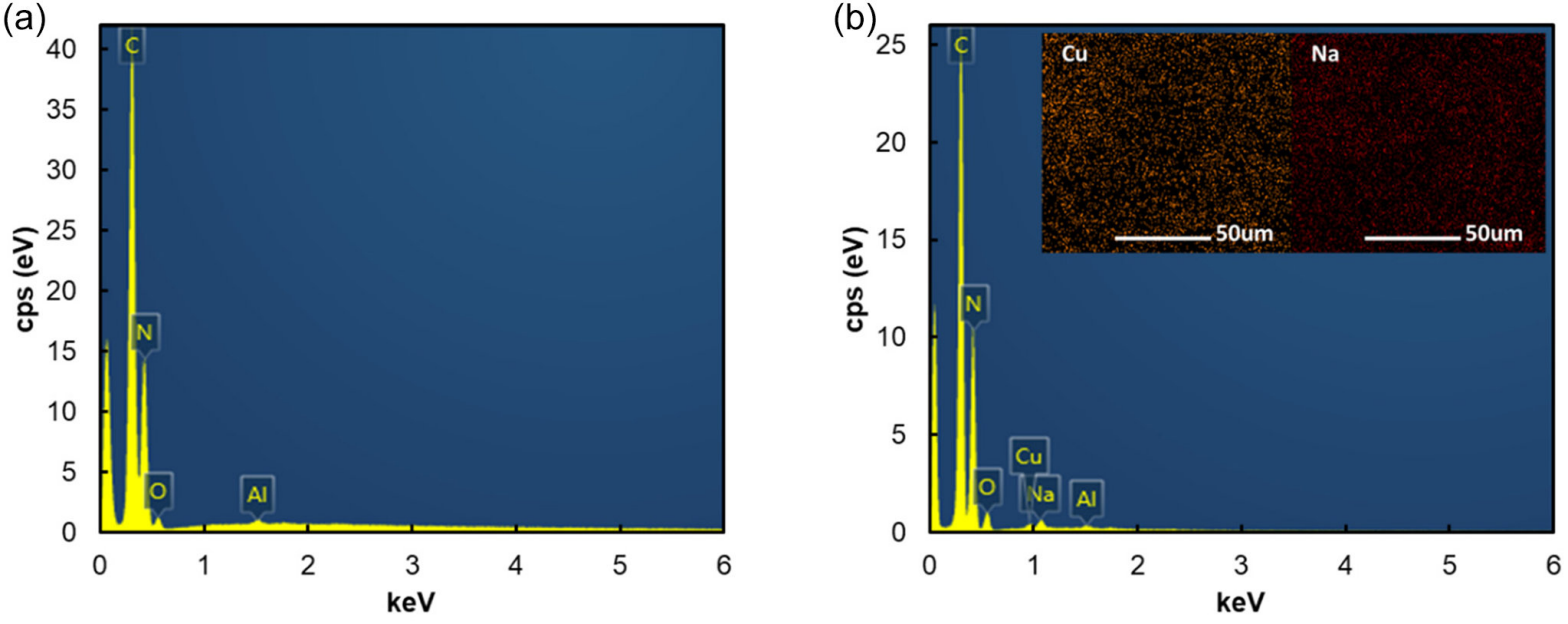
EDX Spectra of (a) g‐C_3_N_4_ and (b) NaCu‐g‐C_3_N_4_. Inset images in (b) show EDX mapping of Cu and Na.

Samples were further examined using SEM (Figure 3) and EDX (Figure 4). Figure 3a–d displays the pristine graphitic carbon nitride and NaCu‐g‐C_3_N_4_ surface SEM morphologies. The synthesized g‐C_3_N_4_, shown in Figure 3a,b, confirmed its characteristic structure [46]. SEM analysis of NaCu‐g‐C_3_N_4_ revealed no visible morphological change after the codoping process (Figure 3c,d), confirming that the g‐C_3_N_4_ backbone was not altered by the introduction of Na and Cu impurities into the lattice of the polymeric semiconductor material. The catalyst's elemental surface composition, validated by EDX (Figure 4a,b), showed the presence of Na and Cu only in the codopped photocatalyst, indicating successful surface modification. The EDX mapping image (Figure 4b) showed Na and Cu elements uniformly distributed over the catalyst's surface.

**FIGURE 5:**
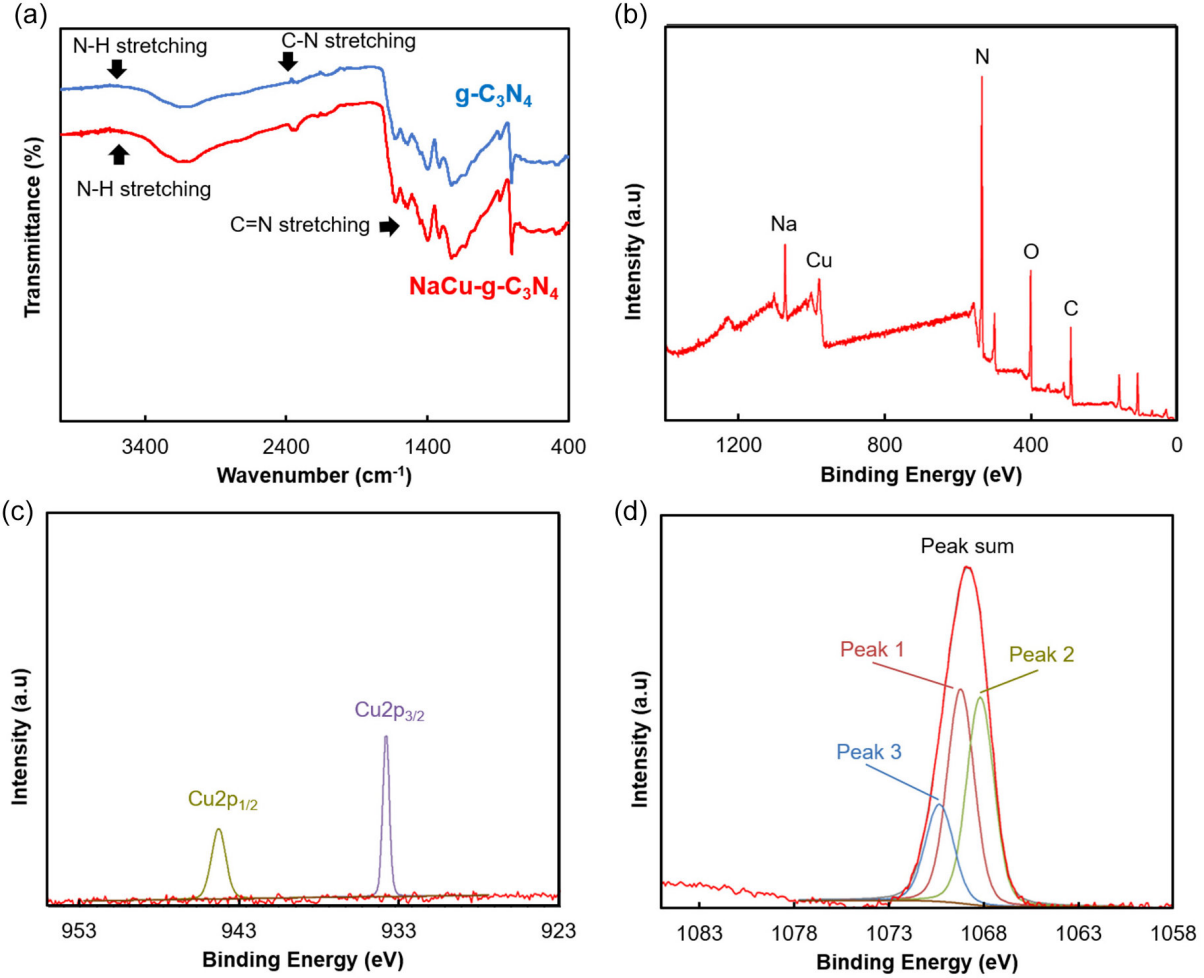
Molecular surface characterization of the synthesized photocatalysts. (a) FTIR spectra of both g‐C_3_N_4_ and NaCu‐g‐C_3_N_4_; (b) XPS spectra of NaCu‐g‐C_3_N_4_ showing all elements; (c) XPS spectra for different oxidations states of Cu in NaCu‐g‐C_3_N_4_; and (d) XPS spectra of Na in NaCu‐g‐C_3_N_4_.

Infrared spectrum analysis of the photocatalysts with FTIR provided further insights into the structural and chemical modifications induced by codoping. The bending mode peak at 804 cm^−1^, characteristic of the tri‐s‐triazine rings, remained prominent (Figure 5a), indicating that the fundamental g‐C_3_N_4_ framework was preserved during the codoping process [47]. However, the shift and alteration in the peaks within the 1230–1407 cm^−1^ range suggested changes in the chemical environment of the carbon nitride heterocycles [48]. These changes can be linked to the introduction of Na and Cu dopants, which may create localized electronic and structural distortions [49, 50]. Such distortions from bond reconfigurations or the incorporation of dopant‐induced defects have the potential to modify the electron density around the carbon nitride bonds, thereby creating conductive pathways that enhance electron transfer to the pollutant [51].

Chemical analysis with XPS validated the presence of Na and Cu in the codoped catalyst (Figure 5b). An oxygen peak was visible at around 530 eV, whereas the nitrogen and carbon peaks were detected around 398.0 and 287.0 eV, respectively. A high‐resolution XPS spectrum of Cu2p (Figure 5c) exhibited peaks with binding energies of 933.0 and 953.0 eV, corresponding to Cu2p3/2 and Cu2p1/2, respectively [52]. These findings confirmed that Cu has been successfully doped onto g‐C_3_N_4_, with Cu atoms in a divalent oxidation state. The presence of Cu^2+^ is important as it can act as an electron acceptor in photocatalytic processes, facilitating charge separation and enhancing the generation of reactive species [53]. Figure 5d presents a high‐resolution XPS spectrum of the Na 1*s* core level, showing a main peak at ≈1070 eV—characteristic of oxidized sodium—with three deconvoluted components (Peaks 1–3) corresponding to oxidation states of chemical environments of Na [54]. Oxidized sodium can influence the electronic structure of the g‐C_3_N_4_ framework by creating localized electronic states or defects that improve light absorption and charge carrier mobility [55].

**FIGURE 6:**
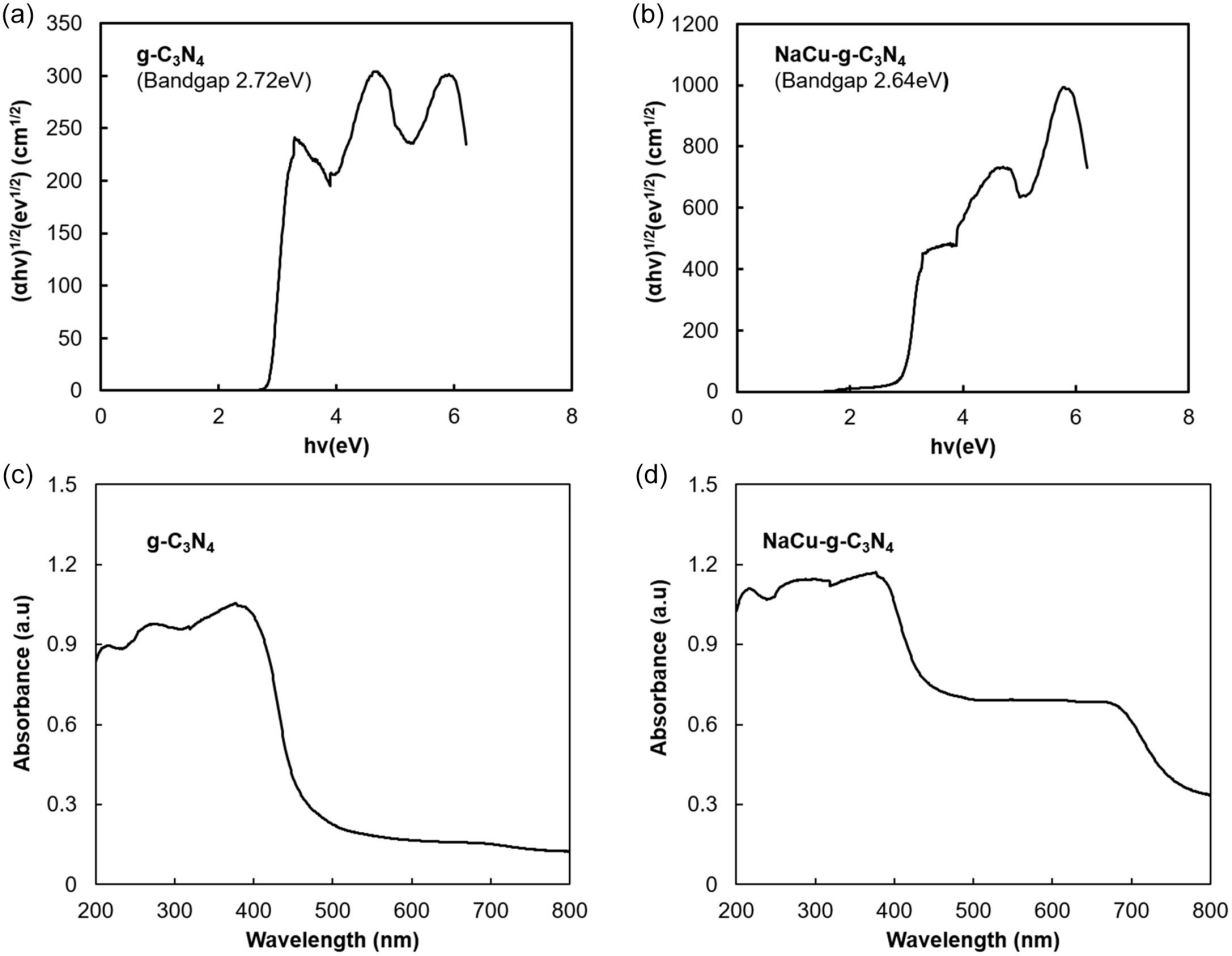
Tauc's plot for bandgap calculation for (a) g‐C_3_N_4_ and (b) NaCu‐g‐C_3_N_4_ and UV–Vis absorbance spectra for (c) g‐C_3_N_4_ and (d) NaCu‐g‐C_3_N_4_.

UV–Vis analysis revealed the ultraviolet–visible diffuse reflectance spectra of g‐C_3_N_4_ and NaCu‐g‐C_3_N_4_, enabling the study of their optical absorption capabilities, including the bandgap. As shown in Figure 6a,b, the bandgap energy (*E*_g_) was calculated from the intercept of the tangents of the plots of (*αhv*)^1/2^ against photon energy [50]. For g‐C_3_N_4_ and NaCu‐g‐C_3_N_4_, the optically determined bandgaps were ≈2.73 and 2.64 eV, respectively. A wider bandgap can result in greater redox potential and longer charge carrier lifetimes, which can be achieved by moving the conduction and valence bands in opposing directions [51]. Figure 6c demonstrates NaCu‐g‐C_3_N_4_ exhibited a strong adsorption band response in the visible‐light range from 400 to 650 nm. NaCu‐g‐C_3_N_4_ showed the maximum absorption in the visible range due to its smaller bandgap (*E*_g_ = 2.64 eV), indicating generation of light‐induced charged species, leading to the excitation of electrons in the presence of visible light [52]. The codoped material enhanced absorption in the visible region, likely boosting photocatalytic activity. This was subsequently evaluated using the visible‐light photooxidation of the recalcitrant contaminant Iopamidol.

Finally, zeta potential and electrical conductivity measurements were performed using a Zetasizer Ultra analyzer (Malvern Panalytical, UK). For each analysis, aqueous suspensions of NaCu‐g‐C_3_N_4_ and g‐C_3_N_4_ were prepared in deionized water, ultrasonicated for 10 min to ensure homogeneous dispersion, and subsequently equilibrated for 5 min prior to measurement. The pH of the suspensions was adjusted using 1 M HCl and 1 M NaOH. All measurements were carried out at 25°C using folded capillary cells, and each reported value represents the mean of three independent determinations. The electrical conductivity of the suspensions was recorded simultaneously under identical experimental conditions to assess the ionic environment of the photocatalyst systems.

### 3.2 Visible‐Light Photocatalytic Oxidation of Iopamidol with NaCu‐g‐C_3_N_4_ and the Role of pH

**FIGURE 7:**
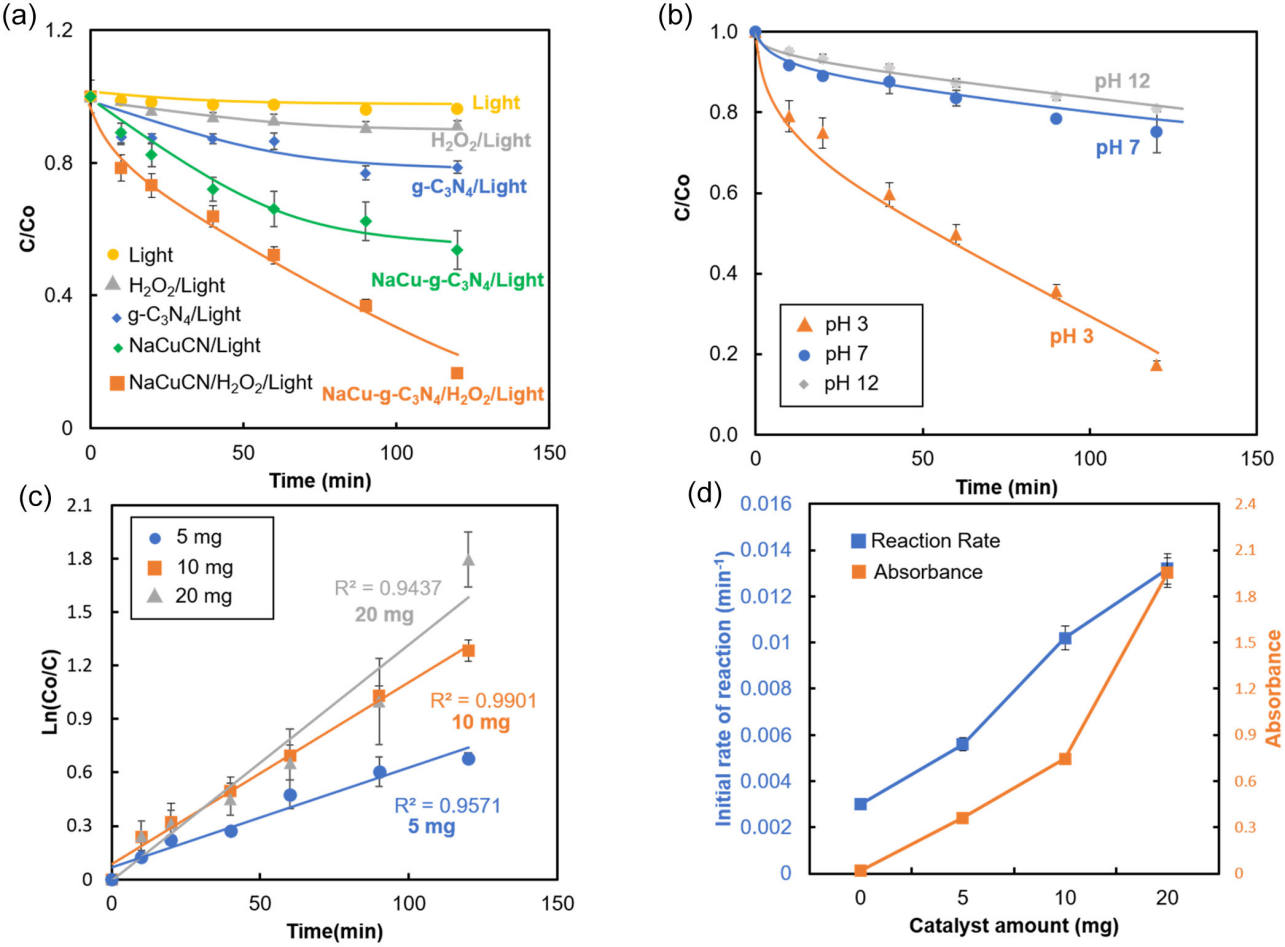
Visible‐light photodegradation of Iopamidol. (a) 10 mg L^−1^ Iopamidol under different systems at pH 3, 5 mM H_2_O_2_ with 20 mg of catalyst; (b) effect of pH in the degradation of 10 mg L^−1^ Iopamidol (20 mg NaCu‐g‐C_3_N_4_, 5 mM H_2_O_2_); (c) kinetic analysis showing visible‐light degradation of 10 mg L^−1^ Iopamidol using NaCu‐g‐C_3_N_4_ at pH 3 and 5 mM H_2_O_2_ follows pseudo‐first order at different catalyst amounts; (d) effect of mass of NaCu‐g‐C_3_N_4_ on initial rate of degradation of 10 mg L^−1^ Iopamidol (pH 3, 5 mM H_2_O_2_). Error bars represent  ±1 standard deviation, resulting from at least three independent replicas.

Photodegradation experiments were conducted under visible‐light irradiation using an LED source at pH 3, both in the presence and absence of H_2_O_2_. In the absence of the photocatalyst, exposure to light and 5 mM H_2_O_2_ resulted in only 18% degradation of Iopamidol after 2 h (Figure 7a). When pristine g‐C_3_N_4_ was introduced under the same conditions, the degradation efficiency remained modest. In contrast, NaCu‐g‐C_3_N_4_ alone achieved 55% degradation, demonstrating superior photocatalytic activity. Notably, the combined use of NaCu‐g‐C_3_N_4_ and H_2_O_2_ significantly enhanced the photocatalytic activity, reaching a degradation efficiency of 82.3%, approximately four times higher than the degradation observed with g‐C_3_N_4_ and H_2_O_2_ under identical conditions. The improvement was attributed to the synergetic effects introduced by Na and Cu during the codoping, together with the presence of H_2_O_2_. While H_2_O_2_ generates hydroxyl radicals that contribute to photocatalytic activity, the codoping enhanced visible‐light absorption and increased the surface area, as confirmed by bandgap analysis and BET measurements. Also, H_2_O_2_ can act as an electron acceptor by interacting with the photogenerated electrons (e^−^) from the catalyst surface to produce hydroxyl radicals according to the following reaction [56]

(1) $$H_{2}O_{2}+e^{-}\to \text{•}\mathrm{OH}+{\mathrm{OH}}^{-}$$

Hydroxyl radicals are highly reactive oxidizing agents that can rapidly degrade organic pollutants like Iopamidol into smaller, less harmful compounds [57]. The role of Na and Cu dopants in NaCu‐g‐C_3_N_4_ is critical, as they may create active sites that enhance H_2_O_2_ activation and improve the generation of •OH radicals [58]. Also, the codoping of g‐C_3_N_4_ with Na and Cu can improve electron transfer, as both elements act as electron mediators, effectively facilitating the movement of electrons [59, 60]. Sodium doping can also enhance the hydrophilicity of the catalyst, improving its interaction with water molecules and facilitating the generation of reactive species during photocatalysis [61].

To assess the impact of starting pH of the solution, a series of Iopamidol degradation experiments were performed ranging from pH 3–12. Iopamidol conversion was maximum at pH 3, experiencing a decline upon increasing the pH to 12 (Figure 7b). Low pH environment was therefore more effective for Iopamidol degradation than the basic pH environment. This can be explained by the enhanced formation of hydroxyl radicals under acidic conditions, which are crucial for breaking down pollutants [62]. In acidic environments, the reaction is more favorable and likely to follow the reactions [63]

(2) $$H_{2}O_{2}+H^{+}\to H_{3}O_{2}^{+}$$

(3) $$H_{3}O_{2}^{+}+e^{-}\to \text{•}\mathrm{OH}+H_{2}O$$

The protonation of H_2_O_2_ under acidic conditions enhanced its susceptibility to reduction by photogenerated electrons (e^−^) from the catalyst surface, leading to the generation of hydroxyl radicals for the degradation of Iopamidol [64]. The low pH also stabilized the charge separation in the photocatalyst, reducing recombination of photogenerated electrons and holes (h^+^), which is critical for maintaining high radical production [65].

**FIGURE 8:**
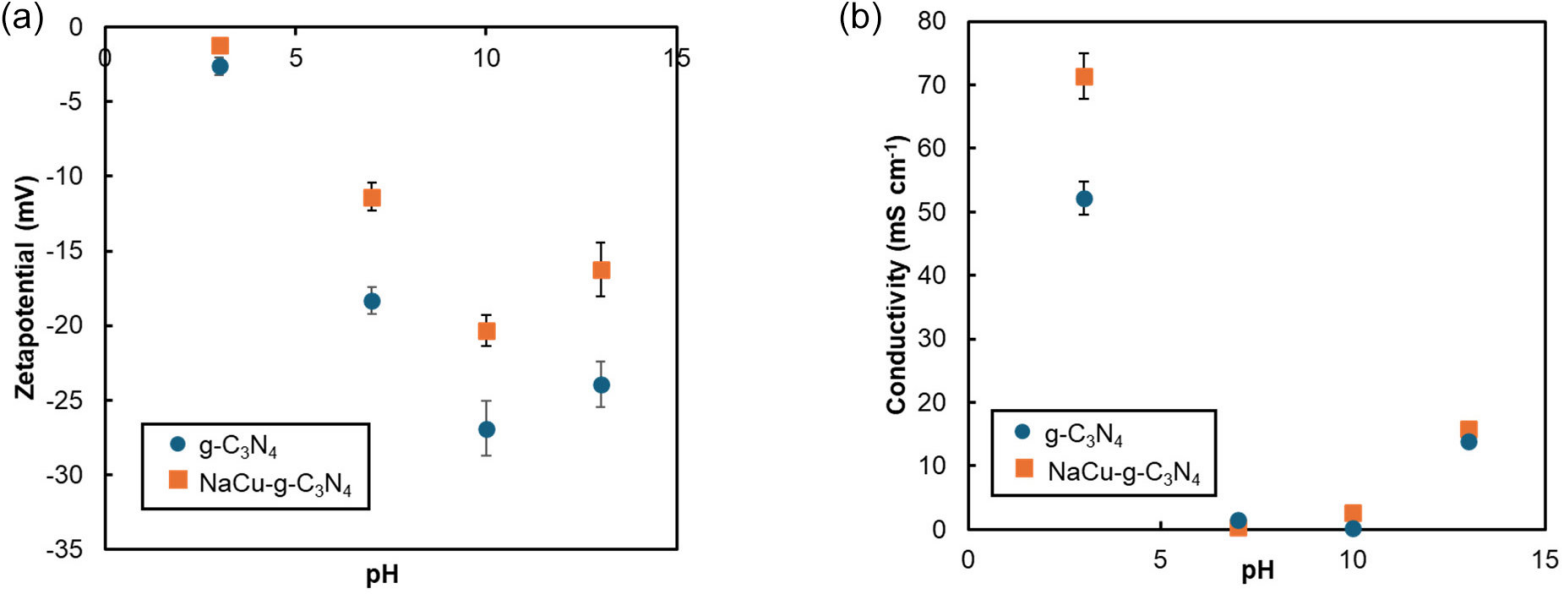
(a) Electrical characterization of NaCu‐g‐C_3_N_4_ and g‐C_3_N_4_ solutions—Zeta potential measurements (20 mg NaCu‐g‐C_3_N_4_, 20 mg g‐C_3_N_4_, and no addition of pollutant nor H_2_O_2_); (b) conductivity measurements (20 mg NaCu‐g‐C_3_N_4_, 20 mg g‐C_3_N_4_, and no addition of pollutant nor H_2_O_2_). Error bars represent ±0.5 standard deviation, resulting from at least three independent replicas.

Analysis using zeta potential and conductivity measurements (Figure 8a,b) provided further valuable insights into the performance of NaCu‐g‐C_3_N_4_ and g‐C_3_N_4_ photocatalysts under varying pH conditions. Pristine g‐C_3_N_4_ exhibited a lower zeta potential at acidic pH compared to NaCu‐g‐C_3_N_4_, which could be due to particle aggregation, as shown in Figure S1, thereby reducing the available active surface area and limiting its interaction with light and Iopamidol [66]. The codoping of Na and Cu in NaCu‐g‐C_3_N_4_ induced surface modifications that enhance the zeta potential at acidic conditions [67]. A high zeta potential at low pH indicates increased electrostatic repulsion between particles, which prevents aggregation and promotes better dispersion of NaCu‐g‐C_3_N_4_ in the solution [68]. This improved dispersion of the NaCu‐g‐C_3_N_4_ particles results in a larger surface area to interact with light and Iopamidol [69]. Iopamidol has a PKa value of 10.7, and therefore remains in its protonated form under acidic conditions [70]. As a result, at pH 3, the surface of the codoped catalyst carries a charge opposite to that of the pollutant, which enhances the attraction between them. This stronger interaction promotes adsorption of the pollutant onto the catalyst surface, thereby facilitating effective charge transfer processes, which are relevant for photocatalytic activity [71] . These properties combined synergistically to deliver the enhanced photocatalytic activity of NaCu‐g‐C_3_N_4_ for visible‐light oxidation of Iopamidol.

To study the effect of pH on the morphology and dispersion of NaCu‐g‐C_3_N_4_ catalyst, samples were also prepared and analyzed at pH 3, 7, and 12. At pH 3, microscopy revealed that the catalyst particles were smaller and more uniformly dispersed, whereas at neutral and basic conditions (pH 7 and 12), the particles appeared larger and more aggregated, as shown in Figure S2. This enhanced dispersion at low pH is likely to increase surface area and improve active site accessibility, contributing to higher photocatalytic efficiency in the degradation of Iopamidol [72].

Since the dispersion and surface characteristics of a catalyst can be influenced by solution chemistry, additional experiments were performed to examine the effect of ionic strength on photocatalytic activity. Sodium chloride was used to adjust the ionic strength of the solution, resulting in a high conductivity of 72 mS/cm. Despite the elevated conductivity, NaCu‐g‐C_3_N_4_ exhibited minimal photocatalytic degradation of Iopamidol under these conditions, as shown in Figure S3a. This suggests that higher conductivity did not influence the degradation performance of the NaCu‐g‐C_3_N_4_/H_2_O_2_ system in the photooxidation of Iopamidol. The stability of the ionic environment, as shown in Figure S3b, confirmed that while sodium chloride enhanced conductivity, it did not contribute meaningfully to photocatalytic activity, underscoring that conductivity is not a primary factor in the degradation process.

Photocatalytic degradation as shown by initial reaction rate (Figure 7c,d) was found to increase with increasing concentration of NaCu‐g‐C_3_N_4_ in the range tested (0–20 mg), at pH 3. Although photocatalytic activity is expected to increase with an increase in surface area, this also means that a higher concentration of photocatalytic particles can reduce the light penetration distance and therefore the overall reaction rates. In the absence of the photocatalyst, degradation was minimal (≈4%), likely driven by noncatalytic processes such as photolysis or weak adsorption of Iopamidol to the surface of the photocatalyst. Doubling the catalyst concentration from 5 to 10 mg increased the rate of degradation, indicating that additional catalytic sites contributed modestly to the overall reaction efficiency, as shown in Figure 7c. Higher concentration of photocatalyst particles provides more active sites for pollutant interaction and light absorption, thus enhancing the generation of reactive species [73]. However, at the highest photocatalyst concentration (20 mg), the reaction rate approached a plateau, likely due to limited light penetration caused by increased absorbance and scattering, as also shown in Figure 7d. The use of high concentrations of photocatalytic particles is desirable as it enhances the generation of hydroxyl radicals, critical in breaking down Iopamidol and facilitating redox reactions [74]. With additional photocatalyst particles, a larger portion of the incident light is absorbed, leading to a higher rate of electron–hole pair generation, fundamental for driving photocatalytic reactions [75]. Also, excessive catalyst amounts can lead to light‐shielding effects, where particles block light from reaching others, reducing the efficiency of light utilization and photocatalytic degradation [66, 67]. It was observed that the degradation process followed a pseudo‐first‐order kinetics in all cases, with a rate constant of 0.013 min^−1^ at 20 mg NaCu‐g‐C_3_N_4_ at pH 3 (Figure 7c).

**FIGURE 9:**
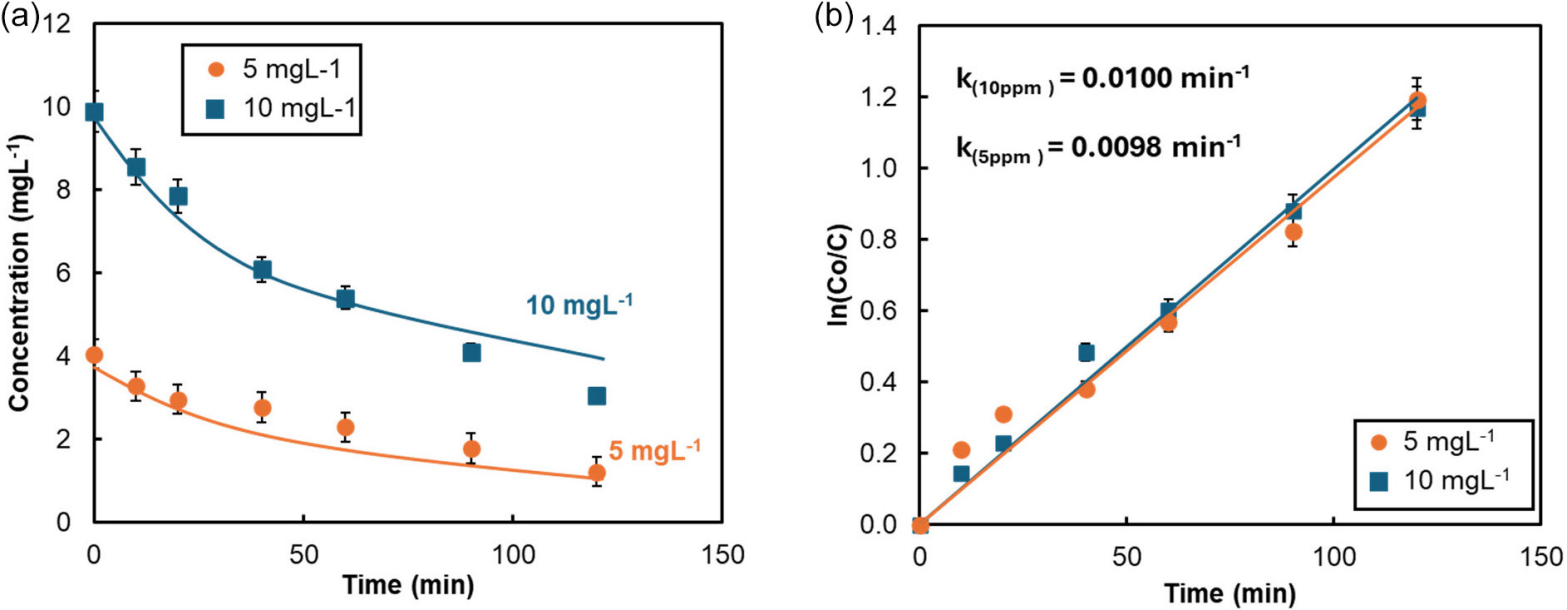
Effect of pollutant (Iopamidol) concentration (a,b) at 5 and 10 mg L^−1^ (pH 3, 5 mM H_2_O_2_, 20 mg of NaCu‐g‐C_3_N_4_), showing how substrate interactions influence photodegradation efficiency. Error bars represent ±0.5 standard deviation, resulting from at least three independent replicas.

When the concentration of Iopamidol was halved from 10 to 5 ppm at pH 3, the kinetic constant remained unchanged (0.0100 vs. 0.0098 min^−1^, respectively, Figure 9a,b). This consistency in the rate constant confirms that the reaction kinetics are truly pseudo‐first order for Iopamidol, as the rate constant in such systems was independent of substrate concentration [76]. The consistent kinetic constant also suggests that the process is not limited by external mass transfer, which would otherwise result in apparent changes in the rate constant at different concentrations [77]. Therefore, the results imply that the degradation of Iopamidol is controlled by the intrinsic surface reaction on the NaCu‐g‐C_3_N_4_ catalyst, where sufficient active sites are available and pollutant transport to the catalyst surface is not rate limiting.

## 4 Conclusions

This study shows that Na and Cu codoping significantly enhances the photocatalytic performance of g‐C_3_N_4_ by reducing the bandgap, improving visible‐light absorption, increasing surface area, and facilitating charge transfer. The resulting NaCu‐g‐C_3_N_4_ achieved 55% degradation of Iopamidol within 120 min under visible LED irradiation at pH 3, compared to 23% for pristine g‐C_3_N_4_, demonstrating the enhanced inherent photocatalytic activity of NaCu‐g‐C_3_N_4_. The addition of 5 mM H_2_O_2_ further boosted performance by generating •OH radicals, increasing degradation efficiency to 82.3%, the highest reported under visible‐light conditions for this pollutant. Acidic conditions (pH 3) enhanced catalyst dispersion (zeta potential: −1.5 mV), increasing active surface area and photocatalytic activity. This work demonstrated a synergistic effect between metal codoping and H_2_O_2_ activation, providing not only an effective strategy for tackling Iopamidol in environmental water but also establishing a foundation for advancing visible‐light‐driven photocatalysis for other emerging contaminants.

## Funding

This study was supported by Engineering and Physical Sciences Research Council (EP/Y003063/1), Royal Society (RGS\R1\221289), Research England.

## Conflicts of Interest

The author declares no conflicts of interest.

## Supporting information

Supplementary Material

## Data Availability

The data will be available in a University of Bath repository with this https://doi.org/10.15125/BATH‐01611.
