# Supplementary material for: Na/Cu‐Doping Engineering of Carbon Nitride for High‐Performance Visible‐Light Degradation of Iopamidol
Source: Small Sci. 2026 Jul 8;6(7):e70341. doi: 10.1002/smsc.70341 (PMC13346769; doi:10.1002/smsc.70341)
Supplement: Supplementary file 1 — Supplementary Material [file SMSC-6-e70341-s001.pdf]

Supporting Information

Na-Cu-Doping Engineering of Carbon Nitride for High-Performance Visible-Light Degradation of Iopamidol

Samuel A. Abey[a,b], Carl Fernandes[a], Emma Emanuelsson[a,c], Nuno M. Reis[a,d], and Antonio J. Exposito[a,b*]

[a] Department of Chemical Engineering, University of Bath, Claverton Down, BA2 7AY UK

[b] Centre for Integrated Materials, Processes & Structures (IMPS), University of Bath, Claverton Down, BA2 7AY UK. Email: ajes22@bath.ac.uk

[c] Centre for Regenerative Design & Engineering for a Net Positive World (RENEW), University of Bath, Claverton Down, BA2 7AY UK.

[d] Centre for Bioengineering and Biomedical Technologies (CBio), University of Bath, Claverton Down, BA2 7AY UK.

S1. Effect of Low pH on dispersion of g-C_3N_4 and NaCu-g-C_3N_4

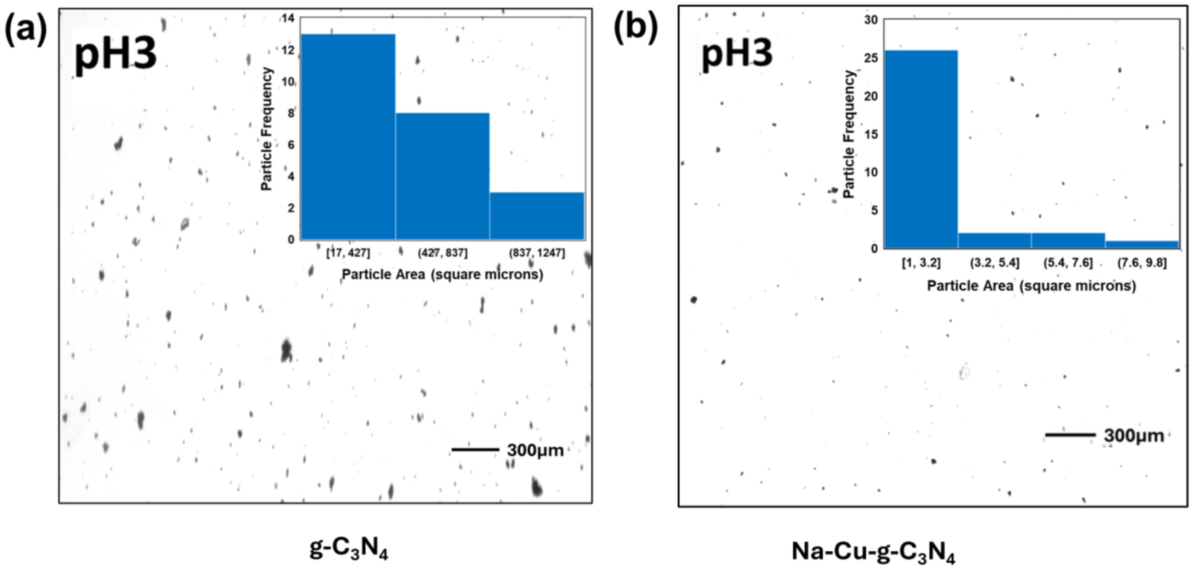

Figure S1. Particle size distribution of (a) g-C_3N_4 and (b) NaCu-g-C_3N_4 in water at pH 3

S2. pH effect on particle size distribution of NaCu-g-C_3N_4

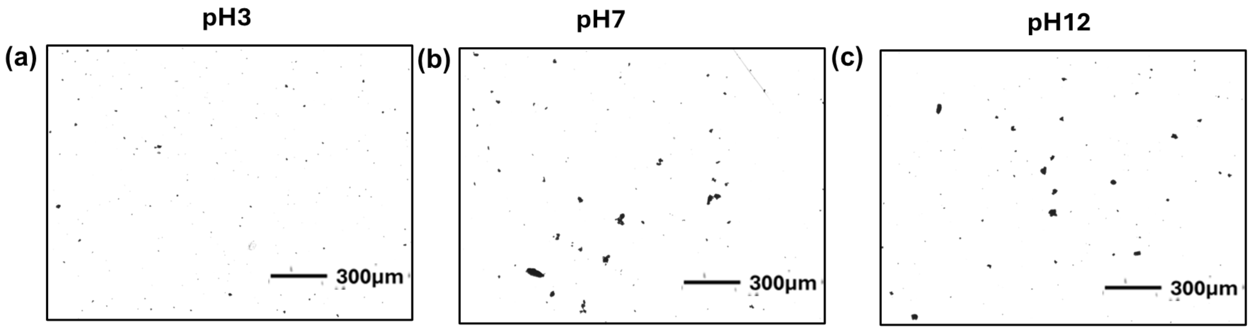

Figure S2. Particle size distribution of NaCu-g-C_3N_4 in water at (a) pH 3; (b) pH 7 and (c) pH 12

S3. Conductivity measurements and NaCl experiments

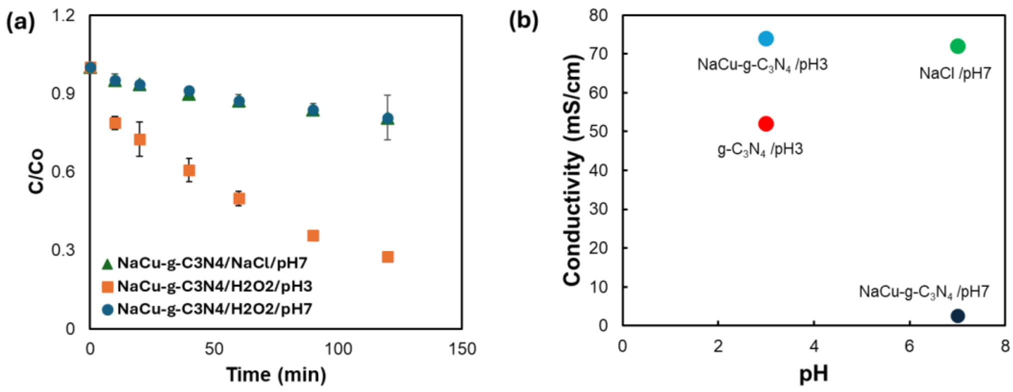

Figure S3. (a) Photocatalytic performance of NaCu-g-C_3N_4/H_2O_2 and in the presence of NaCl, both at pH 7; (b) Conductivity measurements of g-C_3N_4, NaCu-g-C_3N_4, NaCl at different pHs.
